# Former smokers with non‐small‐cell lung cancers: a comprehensive investigation of clinicopathologic characteristics, oncogenic drivers, and prognosis

**DOI:** 10.1002/cam4.764

**Published:** 2016-05-26

**Authors:** Shanbo Zheng, Rui Wang, Yang Zhang, Yunjian Pan, Chao Cheng, Difan Zheng, Yihua Sun, Haiquan Chen

**Affiliations:** ^1^ Department of Thoracic SurgeryFudan University Shanghai Cancer CenterShanghaiChina; ^2^ Department of OncologyShanghai Medical CollegeFudan UniversityShanghaiChina; ^3^ Department of Thoracic SurgeryShanghai Chest HospitalShanghai Jiaotong UniversityShanghaiChina

**Keywords:** EGFR, former smoker, non‐small‐cell lung cancer, prognosis, smoking cessation

## Abstract

The aim of this present investigation was to evaluate the clinicopathologic characteristics, oncogenic drivers, and prognosis of former smokers with non‐small‐cell lung cancer (NSCLC), and to compare them with those of the current and never smokers. This investigation was a single‐institution retrospective study of 2289 NSCLC patients, who were classified as former, current, or never smokers. A collection was made of the clinicopathological characteristics, spectra of well‐identified driver genes and survival rates. The survival rates were compared using log‐rank test, and independent prognostic factors, identified using Cox regression analysis. Of 2289 NSCLC patients, 257 (11.2%) were former smokers; 868 (37.9%), current smokers; and 1164 (50.9%), never smokers. Compared with the current, the former were characterized by older age at diagnosis (64.3y vs. 59.9y; *P* < 0.001), earlier TNM stage (stage I, 47.9% vs. 39.5%; *P* = 0.017), fewer solid predominance in adenocarcinomas (16.2% vs. 29.5%; *P* = 0.005), and more *EGFR* mutation (33.2% vs. 20.7%; *P* < 0.001) but less *KRAS* mutation (6.7% vs. 11.9%, *P* = 0.041). No statistically significant survival differences were observed between the former and current. However, the light former smokers presented favorable overall survival when compared with the light current and heavy former or current (the light former vs. the heavy former, *P* = 0.028; the light former vs. the light current, *P* = 0.048; and the light former vs. the heavy current, *P* = 0.048). Our findings suggest that the former smokers with NSCLCs can have distinctive clinicopathologic characteristics, oncogenic drivers, and prognosis, and they, especially the light former, can benefit from smoking cessation.

## Introduction

Lung cancer is the leading cause of cancer‐related death worldwide, with more than one million people dying from it each year 1. Decades of epidemiologic studies have established cigarette smoking as the most important risk factor for the development of lung cancer 2. These studies indicated that 85–90% of lung cancer cases are associated with cigarette smoking 3.

The association of smoking with lung cancer and subsequent death is irrefutable. Tobacco smoke contains over 60 known carcinogens 4. The relative risk of lung cancer development is estimated to be 10‐ to 30‐fold in long‐term smokers when compared with never smokers 5. For lung cancer patients, smoking history is associated with diagnosis at a later stage, increased operative mortality, higher rate of local recurrence, and worse long‐term survival 6, 7. Furthermore, those who quit smoking develop fewer radiation pneumonitis and infections during radiotherapy, and better response to chemotherapy and targeted therapy 8, 9. Smoking cessation is associated with improved pulmonary function, weight gain, and better quality of life 10.

Although previous studies have already demonstrated the benefits of smoking cessation for lung cancer patients, the reports are limited on associations between detailed smoking statuses and clinical outcomes in former smokers with non‐small‐cell lung cancer (NSCLC). Moreover, few studies have described the clinical and pathologic features and well‐identified driver mutations in the former with NSCLC in comparison with the current and never with NSCLC. In this study, we undertook a comprehensive analysis to further investigate the clinicopathologic features, oncogenic drivers of former smokers and associations between detailed smoking statuses and clinical outcomes.

## Materials and Methods

### Patients

We consecutively enrolled the patients with newly diagnosed non‐small‐cell lung cancer between October 2007 and May 2013 at the Department of Thoracic Surgery of Fudan University Shanghai Cancer Center, Shanghai, China. The history of cigarette smoking were obtained at the patient interview by professional doctors, and the patients were categorized into never smokers, former smokers, and current smokers according to their smoking statuses. The never were defined as those who had smoked less than 100 cigarettes; of the smokers, the former, as those who had quitted smoking 1 year ago or more before diagnosis; and the current, as those who continued their smoking habit at diagnosis or had quitted smoking less than 1 year ago. Pack‐years of smoking were calculated by multiplying the number of packs (one pack containing 20 cigarettes) smoked per day by the number of smoking years. This study was approved by the Institutional Review Board of Fudan University Shanghai Cancer Center, with written informed consent obtained from all patients. Disease recurrence and survival were observed in the follow‐up clinic or obtained by telephone.

### Clinical and pathological variables

The clinical and pathological data collected for analyses covered sex, age at diagnosis, detailed information regarding cigarette smoking such as daily cigarette use, duration of smoking history and quit date if applicable, pathological TNM stage, tumor differentiation, and histological types according to the new IASLC/ATS/ERS multidisciplinary classification 11. Pathological TNM stages were evaluated in accordance with the seventh edition of the lung cancer staging classification system 12.

### Mutation analyses

Comprehensive mutational analyses of *EGFR*,* KRAS*,* HER2*,* BRAF*,* ALK*,* ROS1*,* RET,* and *FGFR* were performed in the patients with NSCLC. Tumor samples resected with curative intent were snap‐frozen in liquid nitrogen at the time of resection and stored in liquid nitrogen. RNA was extracted from tumors or distant histological normal lung as per standard protocols after frozen specimens were dissected into TRIZOL (Life Technologies, Carlsbad, CA). Total RNA samples were reverse transcribed into complementary DNA (cDNA) using RevertAid First Strand cDNA Synthesis Kit (Fermentas, St Leon‐Rot, Germany). *EGFR* (exons 18–22), *KRAS* (exons 2–3), *HER2* (exons 18–21), and *BRAF* (exons 11–15) were amplified by polymerase chain reaction (PCR) using cDNA. Amplified products were analyzed by direct dideoxynucleotide sequencing. *ALK*,* RET*,* ROS1*, and *FGFR* fusions were analyzed by qRT‐PCR plus RT‐PCR and confirmed by FISH as we had previously reported 13, 14, 15, 16. PCR products were directly sequenced in forward and reverse directions. All mutations were verified by analysis of an independent PCR isolate.

### Statistical analysis

Difference in proportions was analyzed using Pearson's chi‐squared test, when no cell of a contingency table had an expected count less than five, or Fisher's exact test, when any cell of a contingency table had an expected count less than five. Relapse‐free survival (RFS) and overall survival (OS) of patients with smoking statuses was estimated through the Kaplan–Meier method. The survival differences between groups were determined via the log‐rank test. Independent prognostic factors were identified through the Cox proportional hazards regression (forward likelihood ratio model). All tests were two tailed, and statistical significance was set as *P* < 0.05. All data were analyzed using SPSS Version 19.0 Software (SPSS Inc., Chicago, IL).

## Results

### Patient characteristics

A total of 2289 NSCLC patients were enrolled in this study, all patients of East Asians. As listed in Table 1, the mean age at diagnosis was 60.0 years (ranging 22–88), and 1374 (60.0%) were male. Of 2289, 257 (11.2%) were former smokers; 1164 (50.9%), never smokers; and 868 (37.9%), current smokers. The mean smoking dosage of smokers was 40 pack‐years, ranging from 0.25 to 240. The patients in the stages I‐IV numbered 1167 (51.0%), 383 (16.8%), 676 (29.6%), and 63 (2.8%), respectively. A diagnosis was made of adenocarcinoma in 1492 (65.2%), of squamous cell carcinoma in 630 (27.5%), of adenosquamous carcinoma in 68 (3.0%) and, of large cell carcinoma in 43 (1.9%). Not otherwise specified (NOS) were 41(1.8%), and a combination of different histology was observed in 15 (0.7%).

**Table 1:** Patient clinicopathologic characteristics and mutation profile

Character	Total	Former smoker	Current smoker		Never smoker	
	No. (*n* = 2289)	No.(*n* = 257)	No.(*n* = 868)	P(former vs. current)	No.(*n* = 1164)	P(former vs. never)
Age at diagnosis	2289					
Mean (range), y	60.0 (22‐88)	64.3 (32‐87)	59.9 (34‐82)	<0.001	59.1 (22‐84)	<0.001
≤60 years	1133 (49.5%)	81 (31.5%)	447 (51.5%)	<0.001	605 (52.0%)	<0.001
>60 years	1156 (50.5%)	176 (68.5%)	421 (48.5%)		559 (48.0%)	
Sex	2289					
Male	1374 (60.0%)	247 (96.1%)	853 (98.3%)	0.039	274 (23.5%)	<0.001
Female	915 (40.0%)	10 (3.9%)	15 (1.7%)		890 (76.5%)	
Initial stage	2289					
I	1166 (50.9%)	123 (47.9%)	343 (39.5%)	0.017	700 (60.1%)	<0.001
II/III/IV	1123 (49.1%)	134 (52.1%)	525 (60.5%)		464 (39.9%)	
Histology	2289					
Adenocarcinoma	1492 (65.2%)	117 (45.5%)	359 (41.4%)	0.333	1016 (87.3%)	<0.001
Squamous cell	630 (27.5%)	118 (45.9%)	412 (47.5%)		100 (8.6%)	
AS/L/NOS/other	167 (7.3%)	22 (8.6%)	97 (11.2%)		48 (4.1%)	
Histologic subtypes	1492					
AIS	44 (2.9%)	1 (0.9%)	5 (1.4%)	1.000	38 (3.7%)	0.173
MIA	47 (3.2%)	3 (2.6%)	2 (0.6%)	0.098	42 (4.1%)	0.616
Lepidic	154 (10.3%)	9 (7.7%)	24 (6.7%)	0.710	121 (11.9%)	0.173
Acinar	653 (43.8%)	53 (45.3%)	132 (36.8%)	0.100	468 (46.2%)	0.861
Papillary	186 (12.4%)	20 (17.1%)	45 (12.5%)	0.212	121 (11.9%)	0.110
Solid	238 (16.0%)	19 (16.2%)	106 (29.5%)	0.005	113 (11.1%)	0.104
Micropapillary	23 (1.5%)	2 (1.7%)	5 (1.4%)	0.683	16 (1.6%)	0.709
IMA	77 (5.2%)	4 (3.4%)	19 (5.3%)	0.412	54 (5.3%)	0.378
*EGFR*	1894					
Mutant	902 (47.6%)	65 (33.2%)	139 (20.7%)	<0.001	698 (67.9%)	<0.001
*KRAS*	1881					
Mutant	123 (6.5%)	13 (6.7%)	79 (11.9%)	0.041	34 (3.3%)	0.024
*HER2*	1878					
Mutant	40 (2.1%)	1 (0.5%)	2 (0.3%)	0.538	37 (3.6%)	0.023
*BRAF*	1865					
Mutant	21 (1.1%)	1 (0.5%)	12 (1.8%)	0.318	8 (0.8%)	1.000
*ALK*	1862					
Fusion	76 (4.1%)	5 (2.6%)	14 (2.2%)	0.782	57 (5.6%)	0.085
*RET*	1863					
Fusion	18 (1.0%)	0 (0.0%)	4 (0.6%)	0.579	14 (1.4%)	0.144
*ROS1*	1863					
Fusion	11 (0.6%)	0 (0.0%)	4 (0.6%)	0.579	7 (0.7%)	0.605
*FGFR*	1863					
Fusion	19 (1.0%)	4 (2.1%)	12 (1.8%)	0.769	3 (0.3%)	0.014

AS, adenosquamous carcinoma; L, large cell carcinoma; NOS, non‐small‐cell lung cancer not otherwise specified; AIS, adenocarcinoma in situ; MIA, minimally invasive adenocarcinoma; IMA, invasive mucinous adenocarcinoma.

In lung adenocarcinoma, the most common histologic subtype was acinar predominant (43.8%), followed by solid predominant (16.0%), papillary predominant (12.4%), lepidic predominant (10.3%), and micropapillary predominant (1.5%). Other types included adenocarcinoma in situ (AIS; 2.9%), minimally invasive adenocarcinoma (MIA; 3.2%), and invasive mucinous adenocarcinoma (IMA; 5.2%).

### Differences in clinicopathological features and driver mutations between former, never, and current smokers

A collection was made of the clinicopathological features of the former smokers, which were compared with those of the current and never. The former were diagnosed at an older age than the current and never (64.3year vs. 59.9year, *P* < 0.001; 64.3year vs. 59.1year, *P* < 0.001, respectively). When compared with the current, fewer male patients were defined as the former (96.1% as former vs. 98.3% as current; *P* = 0.039). The former produced an earlier TNM stage than the current (stage I: 47.9% as former vs. 39.5% as current; *P* = 0.017). The former were more likely to develop squamous cell carcinoma than the never, as in the same case of the current.

An analysis was made of the associations between smoking states and histologic subtypes in adenocarcinoma. Fewer solid predominant adenocarcinomas were detected in the former than in the current (16.2% as former vs. 29.5% as current, *P* = 0.005; 16.2% as former vs. 11.1% as never, *P* < 0.104). No differences were observed in other histologic subtypes between the former and the never or the current.

**Figure 1:**
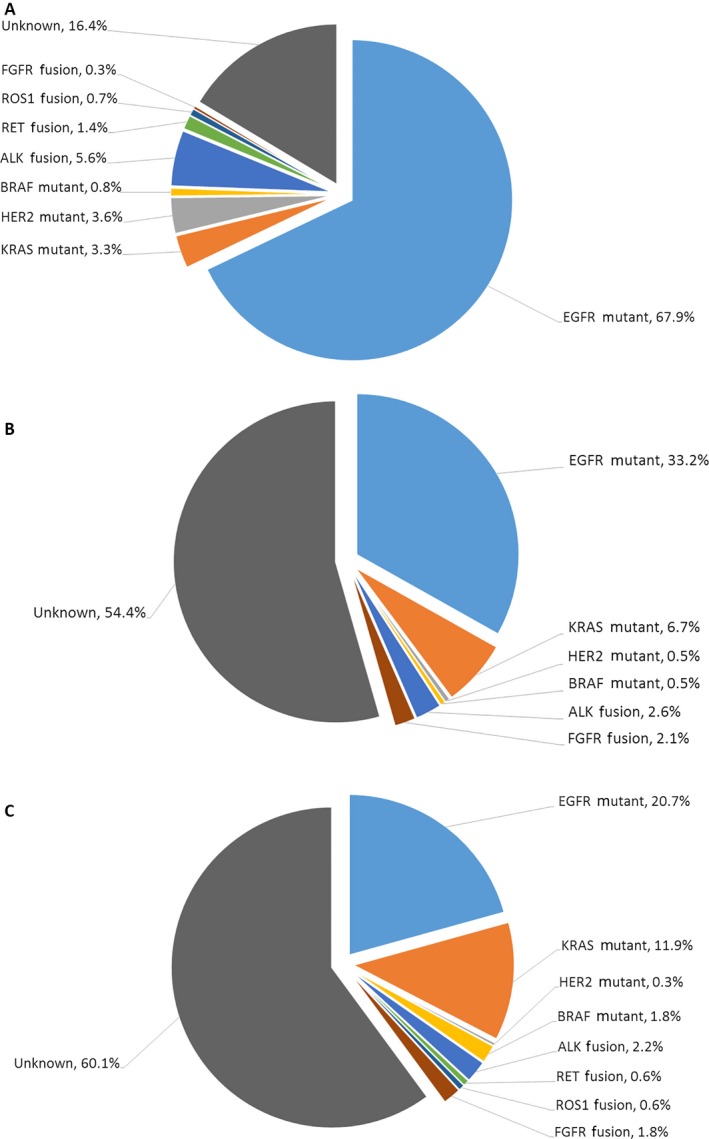
Mutation profiles for never smokers, former smokers, and current smokers with non‐small‐cell lung cancers (NSCLCs). (A) Never smokers. (B) Former smokers. (C) Current smokers.

Mutational analyses were performed in approximately 1800 patients (Fig. 1). Of the former, 33.2% had *EGFR* kinase domain mutations (including 32 exon 19 deletions, 31 L858R, and two other mutations); 6.7%, *KRAS* (including two G12A, three G12C, one G12D, three G12V, one L19F, one R41M, and two Q61H); 0.5%, *HER2* (one exon 20 insertion mutation); and 0.5%, *BRAF* mutations (one L485S). *ALK* rearrangement was detected in the patients by 2.6% (five *EML4*‐*ALK* fusions), and *FGFR* fusion, in those by 2.1% (four *FGFR3‐TACC3* fusions), whereas *RET* and *ROS1* fusion were not found in the former. Interestingly for *EGFR* and *KRAS*, the most common mutations in NSCLC, the mutation profile of the former fell just between the never and the current (*EGFR*: 33.2% as former vs. 20.7% as current, *P* < 0.001; 33.2% as former vs. 67.9% as never, *P* < 0.001; *KRAS*: 6.7% as former vs. 11.9% as current, *P* = 0.041; 6.7% as former vs. 3.3% as never, *P* = 0.024). As in the same case of the current, the former carried fewer *HER2* mutations, but more *FGFR* fusions than the never. As to *BRAF*,* ALK*,* RET,* and *ROS1*, no significant differences were found between the former and the current or the never.

### Univariate analysis of the clinicopathologic variables on survival outcomes

**Figure 2:**
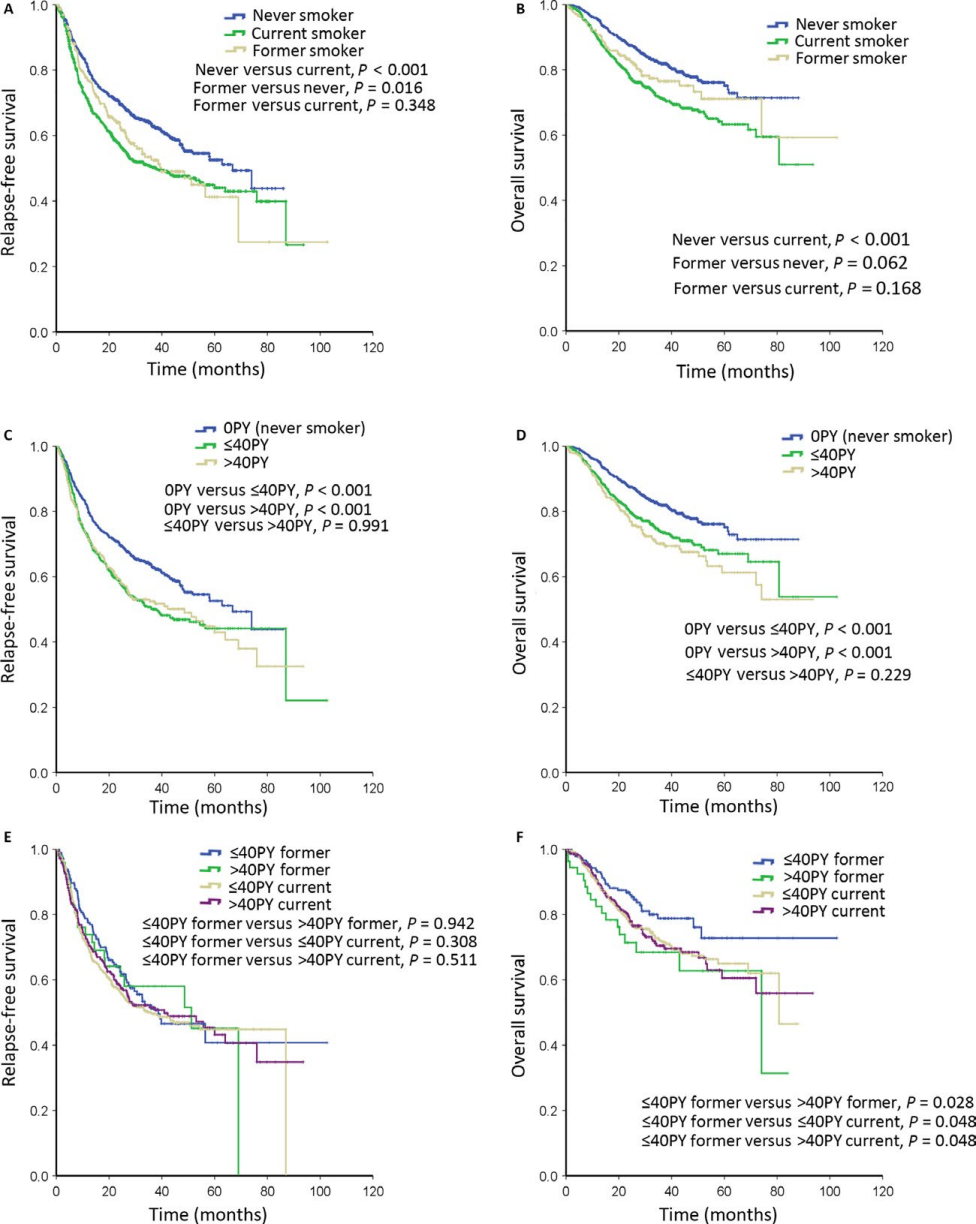
Kaplan–Meier survival curves for relapse‐free survival (RFS) and overall survival (OS) in NSCLC patients. (A) RFS according to smoking status. (B) OS according to smoking status. (C) RFS according to smoking dosage. (D) OS according to smoking dosage. (E) RFS according to combined parameters of smoking status and dosage. (F) OS according to combined parameters of smoking status and dosage. NSCLC, non‐small‐cell lung cancer.

The median follow‐up period was 29.5 months (ranging 1–103 months). The log‐rank test on the Kaplan–Meier survival analysis demonstrated that although there were differences between the former and current in terms of the median RFS and OS, they were not statistically significant (Fig. 2A and B, RFS; median survival: 40 vs. 38 months; log rank: *P* = 0.348; OS: median survival not reached, log rank: *P* = 0.168). Additionally, the never were found to have significantly longer RFS and OS than the current (Fig. 2A and B, RFS; median survival: 67 vs. 38 months; log rank: *P* < 0.001; OS: median survival not reached, *P* < 0.001).

To determine what patients could benefit more from smoking cessation, we focused on detailed smoking information of the former, intending to identify the risk factors for recurrence and mortality. According to the age of smoking cessation before or after 57 as the median, the years of smoking cessation lasting longer or shorter than 5 years as the median duration, and the smoking dosage over or less than 40 pack‐years as the median dosage, the former were divided into different subgroups. The univariate analysis identified that the more advanced initial stage was associated with recurrence, and that smoking dosage over 40 pack‐years, advanced initial stage, *EGFR* wildtype and *FGFR* fusion were associated with mortality (Table 2).

**Table 2:** Univariate analysis of the clinicopathologic variables on survival outcomes in former smokers

Variable	Category	RFS			OS		
		HR	95% CI	*P*‐Value	HR	95% CI	*P*‐Value
Clinicopathologic variables							
Age	>60 years vs. ≤60 years (Ref.)	1.059	0.710–1.577	0.780	1.324	0.765–2.291	0.316
Sex	Female vs. male (Ref.)	0.557	0.176–1.764	0.320	0.359	0.050–2.603	0.311
Age of smoking cessation	>57 years vs. ≤57 years (Ref.)	0.761	0.510–1.135	0.180	1.067	0.619–1.838	0.816
Years of smoking cessation	Quit≤5 years vs. > 5 years (Ref.)	0.765	0.512–1.144	0.192	1.024	0.593–1.766	0.933
Smoking dosage	PY>40 vs.PY≤40 (Ref.)	1.018	0.628–1.651	0.942	1.778	1.054–3.001	0.031
Initial stage	II/III/IV vs. I (Ref.)	3.180	2.503–4.927	<0.001	4.040	2.073–7.872	<0.001
Histology	Adenocarcinoma (Ref.)						
	Squamous cell	1.988	0.770–5.129	0.155	0.967	0.284–3.286	0.957
	AS/L/NOS/other	1.580	0.609–4.099	0.347	1.768	0.538–5.817	0.348
Driver genes							
*EGFR*	Mutant vs. wild type (Ref.)	1.206	0.781–1.861	0.399	0.288	0.129–0.641	0.002
*KRAS*	Mutant vs. wild type (Ref.)	0.705	0.286–1.741	0.449	0.503	0.121–2.095	0.346
*HER2*	Mutant vs. wild type (Ref.)	5.692	0.774–41.839	0.088	3.123	0.492–22.713	0.261
*BRAF*	Mutant vs. wild type (Ref.)	—	—	—	—	—	—
*ALK*	Fusion vs. wild type (Ref.)	1.603	0.393–6.535	0.510	1.069	0.147–7.769	0.947
*RET*	Fusion vs. wild type (Ref.)	—	—	—	—	—	—
*ROS1*	Fusion vs. wild type (Ref.)	—	—	—	—	—	—
*FGFR*	Fusion vs. wild type (Ref.)	0.963	0.134–6.950	0.970	4.774	1.137–20.046	0.033

AS, adenosquamous carcinoma; L, large cell carcinoma; OS, overall survival; NOS, non‐small‐cell lung cancer not otherwise specified.

### Multivariate analysis of the clinicopathologic variables on survival outcomes in former smokers

As indicated by the Cox proportional hazards models (Table 3), the multivariate analysis was adjusted for age, initial stage, age of smoking cessation, years of smoking cessation, smoking dosage, *EGFR,* and *FGFR*. It was found that the late initial stage was a significant and independent risk factor for relapse, while such a stage and *EGFR* wild‐type status were significant and independent risk factors for worse overall survival.

**Table 3:** Multivariate analysis of the clinicopathologic variables on survival outcomes in former smokers

Variable	Category	RFS			OS		
		HR	95% CI	P‐Value	HR	95% CI	*P*‐Value
Age	>60 years vs. ≤ 60 years (Ref.)	1.587	0.871–2.890	0.131	2.026	0.852–4.819	0.110
Age of smoking cessation	>57 years vs. ≤ 57 years (Ref.)	0.816	0.471–1.413	0.468	0.783	0.362–1.693	0.534
Years of smoking cessation	Quit≤5 years vs. > 5 years (Ref.)	0.838	0.511–1.374	0.483	0.843	0.437–1.626	0.609
Smoking dosage	PY>40 vs.PY≤40 (Ref.)	1.000	0.585–1.708	0.999	1.542	0.852–4.819	0.110
Initial stage	II/III/IV vs. I (Ref.)	3.432	2.133–5.525	<0.001	4.643	2.211–9.749	<0.001
*EGFR*	Mutant vs. wild type (Ref.)	1.207	0.749–1.947	0.439	0.294	0.127–0.680	0.004
*FGFR*	Fusion vs. wild type (Ref.)	0.814	0.109–6.093	0.841	3.595	0.778–16.608	0.101

RFS, Relapse‐free survival; OS, overall survival.

### Survival outcomes of cigarette smoking status and dosage

As smoking dosage was an important prognosis factor in the former, we further analyzed the association between smoking dosage and outcomes in all patients, who were divided into three groups: never smokers (0 pack‐years), light smokers (≤40 pack‐years), and heavy smokers (>40 pack‐years). It was found that the never presented more significant RFS and OS than the light and heavy (Fig. 2C & D; RFS: never vs. light, *P* < 0.001; never vs. heavy, *P* < 0.001; OS: never vs. light, *P* < 0.001; never vs. heavy, *P* < 0.001). However, the light did not show significant differences when compared with the heavy (RFS: light vs. heavy, *P* = 0.991; OS: light vs. heavy, *P* = 0.229).

Combining the two important groups of smoking information: smoking status and smoking dosage, we found out that the light former had favorable overall survival than the heavy former, the light current, and the heavy current (Fig. 2F; light former vs. heavy former, *P* = 0.028; light former vs. light current, *P* = 0.048; light former vs. heavy current, *P* = 0.048).

## Discussion

Over the past decades, studies have proved that patients with lung cancer can benefit from smoking cessation; however, there is a death of literature on the clinicopathological characteristics of the former smokers with NSCLC. Previous studies have identified a number of molecularly distinct subsets of lung cancer characterized by different oncogenes 17, 18, 19. Researchers have found tumor mutational frequencies and spectra differences between smokers and nonsmokers, as indicated by the reported differences in the frequency of somatic mutations of *EGFR* and *KRAS* observed between smoking and nonsmoking lung cancer patients 20. However, the mutational spectra of the former smokers with NSCLC have not been reported.

To our knowledge, this study pioneered the comprehensive analysis of well‐identified driver mutations, clinical characteristics, and survival analysis in an Asian cohort of non‐small‐cell lung cancer patients who had quitted smoking. While previous studies have examined the characteristics and outcomes between never and ever smokers, our investigations have focused on NSCLC in former smokers. In this study, we demonstrated that the former smokers were older at diagnosis, showing an earlier TNM stage and harboring more *EGFR* but less *KRAS* mutations than the current. Additionally, we proved that the light former presented favorable overall survival when compared with the light current and the heavy former or the current.

The former with NSCLC were found to benefit from cessation according to the analyses of clinicopathological characteristics, as indicated by the mean age of four years older than that of the current, suggesting a longer lifetime. The findings were in agreement with the data from the NCCN network that former smokers had older age than current smokers 21. When the former had an earlier TNM stage than the current, this was an independent prognosis factor for RFS and OS. In adenocarcinomas, former smokers have been reported to be associated with a lower proportion of solid predominant histology subtype, which is indicative of more aggressive biological features and predicts worse outcomes 22, 23, 24. These clinicopathological characteristics of the former may partly explain their clinical outcomes.

We now understand that NSCLCs are subdivided into different molecular subtypes according to the primary driver genes identified 25. Driver mutations in smoking NSCLCs are different from those in nonsmoking NSCLCs 26, 27. We performed a comprehensive analysis of well‐identified driver genes in the former smokers with NSCLCs, and compared the results with those of the current and never with NSCLCs, demonstrating that the former harbored more *EGFR* but less *KRAS* mutations than the current. It has been reported that NSCLCs with *EGFR* mutations are associated with better outcomes, whereas *KRAS* mutations, with worse outcome 28, 29. Moreover, lung cancer of *EGFR* mutation can be treated with EGFR‐TKI and prolong PFS overall than treated with chemotherapy, especially in those with exon 19 deletions, never smokers and women 30. Our results supported the conclusion that former smokers with NSCLCs may benefit from their specific mutational profiles.

A number of studies have investigated the relationship between smoking and outcome of lung cancer patients, but the results are diverse 7, 20, 21, 31, 32, 33, 34. The present results clearly supported the conclusion that smoking history can exert a negative influence on RFS and OS. Furthermore, the never had favorable RFS and OS rates when compared with those of the current. As to the former, those who had smoked no more than 40 pack‐years showed more significant OS than those who had continued smoking with the same dosage, although smoking dosage is not an independent prognostic factor. Such results emphasized the importance of smoking cessation, even for light smokers.

The present retrospective study had several limitations in the analyses. Smoking extent was reported by the patients themselves without biochemical confirmation; therefore, this could have bias. Additionally, second‐hand tobacco smoke, which has been established as an inducement of lung cancer, was too difficult to quantify exactly and include it in the analyses. Nonetheless, the large number of the patients enrolled for investigation may reduce error in different groups.

In conclusion, NSCLC patients who underwent surgical resection with smoking history can benefit from smoking cessation; former smokers with NSCLCs can age older at diagnosis, presenting an earlier TNM stage and more *EGFR* but less *KRAS* mutations than current ones with NSCLCs; and light former ones can have better long‐term overall survival than heavy former ones as well as light current ones.

## Conflict of Interest

No potential conflicts of interest were disclosed.
